# Early evolution and transmission of GII.P16-GII.2 norovirus in China

**DOI:** 10.1093/g3journal/jkac250

**Published:** 2022-09-19

**Authors:** Luqi Wang, Lei Ji, Hao Li, Deshun Xu, Liping Chen, Peng Zhang, Weibing Wang

**Affiliations:** School of Public Health, Fudan University, Shanghai 200437, China; Huzhou Center for Disease Control and Prevention, Huzhou 313000, China; School of Public Health, Fudan University, Shanghai 200437, China; Huzhou Center for Disease Control and Prevention, Huzhou 313000, China; Huzhou Center for Disease Control and Prevention, Huzhou 313000, China; Huzhou Center for Disease Control and Prevention, Huzhou 313000, China; School of Public Health, Fudan University, Shanghai 200437, China; Key Laboratory of Public Health Safety of Ministry of Education, Fudan University, Shanghai, 200032, China

**Keywords:** norovirus, genomic epidemiology, transmission analysis, phylodynamics, phylogeography, TransPhylo

## Abstract

Norovirus is the most common cause of acute gastroenteritis worldwide. During 2016–2017, a novel recombinant GII.P16-GII.2 genotype of norovirus suddenly appeared and over the next several years became the predominant strain in both China and worldwide. To better understand the origin and diffusion of the GII.P16-GII.2 genotype in China, we conducted molecular evolutionary analyses, including phylodynamics and phylogeography. Moreover, to trace person-to-person transmission of GII.P16-GII.2 norovirus, we applied the novel method, TransPhylo, to a historical phylogeny using sequences obtained from a publicly available database. A time-scaled phylogenetic tree indicated that the time to the most recent common ancestor of the GII.P16-GII.2 major capsid protein (*VP1*) gene diverged from the GII.P2-GII.2 *VP1* gene at 2,001.03 with an evolutionary rate of 3.32 × 10^−3^ substitutions/site/year. The time to the most recent common ancestor of the GII.P16-GII.2 RNA-dependent RNA polymerase region diverged from the GII.P16-GII.4 RNA-dependent RNA polymerase region at 2,013.28 with an evolutionary rate of 9.44 × 10^−3^ substitutions/site/year. Of these 2 genomic regions, *VP1* gene sequence variations were the most influenced by selective pressure. A phylogeographic analysis showed that GII.P16-GII.2 strains in China communicated most frequently with those in the United States, Australia, Thailand, and Russia, suggesting import from Australia to Taiwan and from the United States to Guangdong. TransPhylo analyses indicated that the basic reproductive number (*R*_0_) and sampling proportion (pi) of GII.P16-GII.2 norovirus were 1.99 (95% confidence interval: 1.58–2.44) and 0.76 (95% confidence interval: 0.63–0.88), respectively. Strains from the United States and Australia were responsible for large spread during the evolution and transmission of the virus. Coastal cities and places with high population densities should be closely monitored for norovirus.

## Introduction

Norovirus, a member of the *Norovirus* genus and Caliciviridae family, is the leading cause of acute gastroenteritis worldwide (Ahmed *et al.* 2014). Globally, norovirus causes approximately 677 million cases and 200,000 deaths each year (Pires *et al.* 2015). It is common in both developed and developing countries and causes approximately 50,000 deaths in developing countries each year (Nguyen *et al.* 2017). In general, norovirus circulates in colder weather and causes gastrointestinal symptoms such as vomiting, diarrhea, and abdominal pain. Outbreaks are frequently reported in semiclosed institutions, such as hospitals, nursing homes, and schools (Hall *et al.* 2013).

The genome of norovirus consists of 3 open reading frames (ORFs), ORF1, ORF2, and ORF3, encoding RNA-dependent RNA polymerase (RdRp), major capsid protein (VP1) and minor capsid protein (VP2), respectively (Hardy 2005). Norovirus is highly diverse and is divided into 10 genogroups (GI–GX), which are further divided into at least 40 genotypes (Atmar *et al.* 2019; Li *et al.* 2021). Commonly isolated norovirus genogroups among cases of acute gastroenteritis are GI and GII (Chhabra *et al.* 2019). In the past 2 decades, the GII.4 genotype accounted for the majority of adult outbreaks of gastroenteritis and often swept across the globe (Noel *et al.* 1999; Hoa Tran *et al.* 2013). However, GII.P16-GII.2, a genotype that was rarely reported previously, suddenly reappeared in the winter of 2016–2017 in China, Japan, Germany, France, the United States, and Australia and, within just a few years, became the main genotype in China and other countries (Ao *et al.* 2017; Barreira *et al.* 2017; Niendorf *et al.* 2017; Nagasawa *et al.* 2018). According to monitoring data, most norovirus outbreaks reported in China from 2016 to 2018 were GII.P16-GII.2 (Jin *et al.* 2020).

The genotype distribution, molecular evolution, and amino acid substitution profile of common norovirus genotypes like the GII genogroup group were widely studied in early epidemics (Chan *et al.* 2015; Qiao *et al.* 2017; Ozaki *et al.* 2018, 2019), but few studies have investigated this new recombinant GII.P16-GII.2 norovirus. A previous study concluded that the virus could be traced back to areas around the Pearl River delta in China (Ao *et al.* 2018). Nevertheless, how this genotype evolved worldwide and how it entered China and spread domestically remain unclear. Based on the previous studies, we applied phylodynamics, phylogeography, and TransPhylo methods to better understand the origin, evolution, and transmission process of the GII.P16-GII.2 norovirus and investigated a series of associated epidemic parameters. We found that the GII.P16-GII.2 norovirus may have evolved from GII.P16-GII.4 or previously circulating GII.P16-GII.2 strains, and Guangdong province seems to be the origin of this reemerging GII.P16-GII.2 norovirus in China.

## Materials and methods

### Data set description

All sequences used in this study were downloaded from the National Center for Biotechnology Information (NCBI) database (https://www.ncbi.nlm.nih.gov) (as of October 12, 2021). Sequences used included those with associated geographic location and collection time, and a linear time relationship; in cases of sequences submitted on the same day showing high similarity after sequence alignment, 10 sequences were randomly selected. Ultimately, a total of 164 of 609 GII.2 VP1 complete sequences and 163 of 504 GII.P16 RdRp complete sequences were included in this study. Detailed information is summarized in Supplementary Table 1. A total of 121 complete GII.P16-GII.2 norovirus sequences were downloaded from NCBI (as of October 12, 2021), including 56 sequences from China and 65 sequences from other countries, for analysis of the early origin and diffusion process of GII.P16-GII.2 norovirus in China. Detailed information is summarized in Supplementary Table 2. All norovirus genotypes were also confirmed using an online genotyping tool (https://www.rivm.nl/mpf/typingtool/norovirus) (as of October 12, 2021).

### Bayesian discrete phylodynamics and phylogeography analyses

All sequences were aligned using MUSCLE implemented in MEGA-X (Kumar *et al.* 2018). Regression of root-to-tip distances of sequences was performed using TempEst software (v.1.5.3) (Rambaut *et al.* 2016). After removing sequences that affected the temporal signal, we used RDP4 (v.4.101) to confirm the absence of recombination signals in the dataset (Martin *et al.* 2015). The best-fit HKY + I + G nucleotide substitution model was determined based on the value of LnL in jModelTest (v.2.1.10) (Darriba *et al.* 2012). Time-scaled phylogenetic trees were constructed in BEAST (v.1.8.4) using tip dates (Suchard *et al.* 2018), a strict clock model, and a constant size coalescent model with a Markov chain Monte Carlo (MCMC) sample chain (10^8^ steps with sampling every 1,000 steps). The convergence of parameters was tested using Tracer (v.1.6) software (Rambaut *et al.* 2018), using an effective sample size (ESS) of greater than 200 as an acceptance criterion. A maximum clade credibility tree was constructed using Treeannotator (v.1.8.4) (Drummond and Rambaut 2007), with burn-in of the first 10% of samples, and visualized with FigTree (v.1.4.4) (http://tree.bio.ed.ac.uk/software/figtree). The discrete phylogeography analysis was performed in SpreaD3 (v.0.9.6) software (Bielejec *et al.* 2016). After converting to a keyhole markup language file, we calculated Bayes factors (BFs) in SpreaD3 using a Bayesian stochastic search variable selection (BSSVS) file to obtain statistically significant migration routes. The reliability of the analysis was verified by running the BSSVS independently 3 times, and posterior probability and BF cutoffs were used to define significance.

### Selective pressure analysis

To estimate sites under positive or negative selection pressure in the *VP1* gene among all GII.2 strains and in the *RdRp* region of all GII.P16 strains, we calculated the rates of nonsynonymous (*dN*) and synonymous (*dS*) substitutions at every codon position with Hyphy (v2.3) (Pond *et al.* 2005) using a mixed effects model of evolution (MEME) and fixed effects likelihood (FEL) methods, with a *P*-value threshold of 0.1 (Murrell *et al.* 2012).

### TransPhylo analysis

Because the phylogenetic approach only reveals the relationship between lineages and not transmission links, we used TransPhylo (Didelot *et al.* 2017) to reconstruct the transmission dynamics of the GII.P16-GII.2 genotype. TransPhylo, a software tool implemented as an R package, is designed to reconstruct infectious disease transmission using genomic data based on a combined model of transmission between hosts and pathogen evolution within each host (Didelot *et al.* 2021). Because this tool requires knowledge of Gamma distribution parameters representing the generation time (from infection to transmission), we input previous estimates of 3.35 and 1.09 for shape and scale parameters, respectively (Heijne *et al.* 2009). Transmission is inferred based on MCMC sampling; 500,000 iterations were performed, simultaneously yielding posterior probability, sampling proportion, within-host coalescent rate, and basic reproductive number. Before exploring inference results, we tested convergence and mixing properties based on the trace of each parameter (Supplementary Fig. 1) and ESS, computed with the CODA package. ESS values greater than 500 were accepted. A color-coded tree (Supplementary Fig. 2) containing all information about an outbreak was generated from input of a dated phylogeny, then the transmission tree was extracted from this tree. A series of transmission inference parameters were estimated based on this transmission tree using the TransPhylo method. The probability of direct transmission between cases was computed and visualized using Gephi software (v0.9.2) (Bastian 2009).

## Results

### Phylodynamic analysis of GII.P16-GII.2 norovirus

**Fig. 1.**
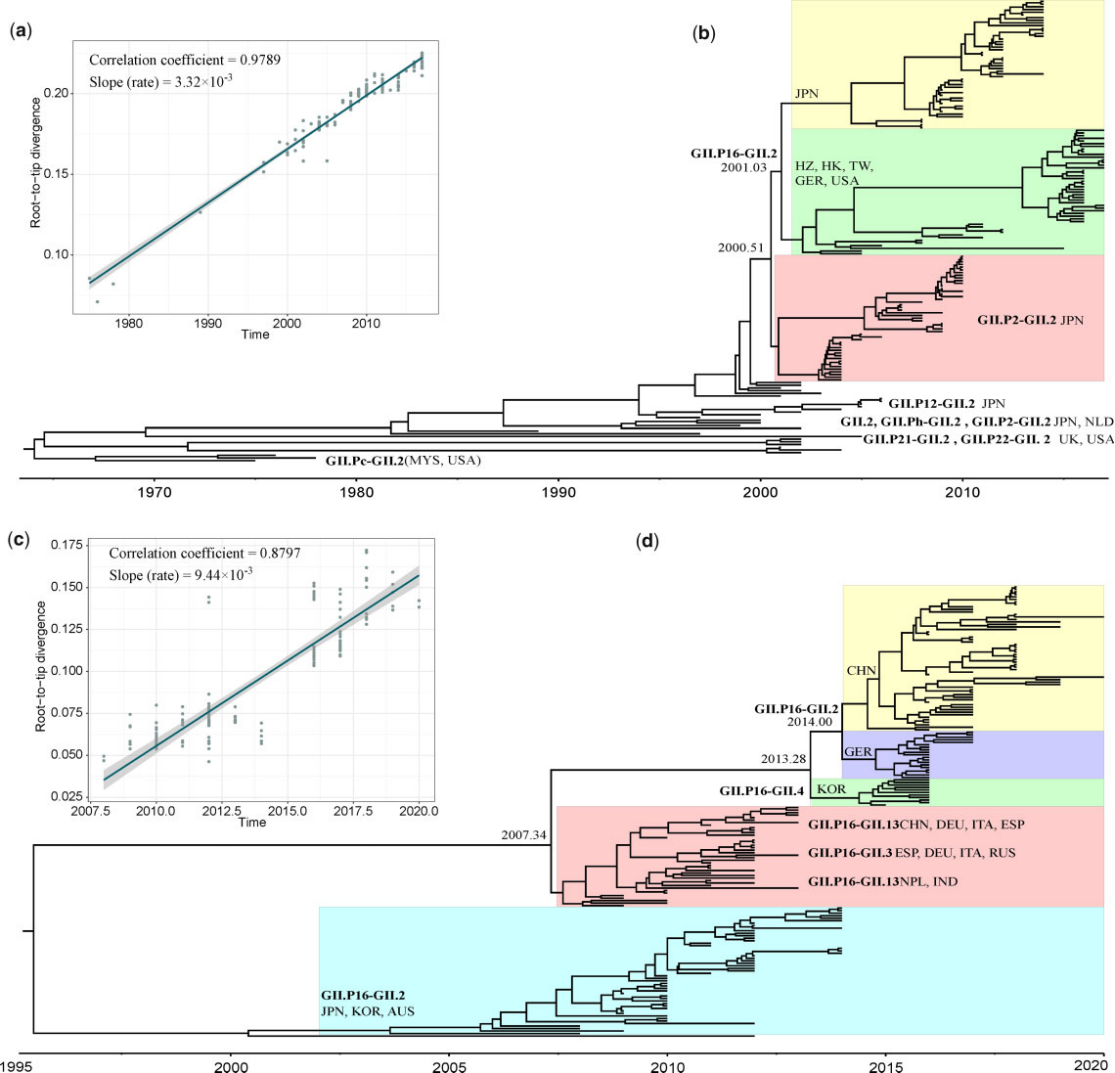
Time-scaled phylogenetic analysis. a) Regression of the root-to-tip genetic distance against year of sampling for GII.2 *VP1* gene sequences. b) Maximum clade credibility tree of the GII.2 *VP1* gene using the Bayesian MCMC method in BEAST. c) Regression of the root-to-tip genetic distance against year of sampling for GII.P16 *RdRp* region sequences. d) Maximum clade credibility tree of the GII.P16 *RdRp* region using the Bayesian MCMC method in BEAST. Country and area abbreviations: AUS, Australia; CHN, China; ESP, Spain; GER, Germany; IND, India; ITA, Italy; JPN, Japan; KOR, South Korea; MYS, Malaysia; NLD, Netherlands; NPL, Nepal; RUS, Russia; UK, the United Kingdom; USA, the United States.

Figure 1a shows that *VP1* gene sequences of GII.2 norovirus evolved at a rate of 3.32 × 10^−3^ [95% confidence interval (CI): 3.30 × 10^−3^–3.33 × 10^−3^] substitutions/site/year. A time-scaled phylogenetic tree based on full-length GII.2 *VP1* gene sequences (Fig. 1b) indicates that there have been 3 major evolutionary clades worldwide since 2000, including 2 clades of GII.P16-GII.2 and 1 clade of GII.P2-GII.2. Of the 2 GII.P16-GII.2 norovirus clades, 1 consists mainly of isolates from Japan, whereas the other consists of isolates from China, Germany, and the United States. The most recent common ancestor (MRCA) of GII.P16-GII.2 occurred at about 2,001.03 [95% highest posterior density (HPD): 2,000.16–2,001.88]. The GII.P2-GII.2 clade consists mainly of isolates from Japan. The MRCA for GII.P16-GII.2 and GII.P2-GII.2 occurred at about 2,000.51 (95% HPD: 1,999.67–2,001.27). Figure 1c shows that GII.P16 has evolved at a rate of 9.44 × 10^−3^ substitutions/site/year (95% CI: 9.16 × 10^−3^–9.72 × 10^−3^). Figure 1d, which shows a time-scaled phylogenetic tree based on the *RdRp* region of GII.P16 norovirus, indicates that GII.P16-GII.2 isolates from China were similar to those from Germany and shared an MRCA at about 2,014.00 (95% HPD: 2,013.19–2,014.68). GII.P16-GII.2 isolates from China and Germany shared an MRCA with GII.P16-GII.4 isolates from Korea at about 2,013.28 (95% HPD: 2,012.29–2,014.06) and then shared an MRCA with GII.P16-GII.13 and GII.P16-GII.3 genotypes at about 2,007.32 (95% HPD: 2,005.5774–2,008.3171). In contrast, GII.P16-GII.2 isolates from China and Germany separated early in 1,995.48 (95% HPD: 1,982.3258–2,002.8254) from those from Japan, Korea, and Australia.

Table 1 shows that 11 and 2 positive selection sites were detected by the MEME method in the GII.2 *VP1* gene and GII.P16 *RdRp* region, respectively. The FEL method detected 3 diversifying positive and 281 purifying selection sites in the GII.2 *VP1* gene and no diversifying positive and 93 purifying selection sites in the GII.P16 *RdRp* region. Sites 61 and 285 were detected as positively selected sites using both methods. These results suggest that, of the 2 genomic regions, sequence variations in the capsid gene (*VP1*) may be under greater selective pressure.

**Table 1.** Positive selection sites in the GII.2 *VP1* gene and GII.P16 *RdRp* sequences.

Sequence	Site	Synonymous rate	Nonsynonymous rate	*P*-Value	MEME LogL	FEL LogL	Amino acid change
VP1 of GII.2	61	0.00	12.15	0.00	−61.49	−59.67	Y → H/C
	78	0.24	69.35	0.02	−37.53	−34.77	Y → H
	180	0.31	133.95	0.00	−41.23	−34.30	P → S/L
	285	0.00	1.13	0.01	−39.29	−39.29	P → A/G/S/T
	291	2.59	48.49	0.04	−52.76	−47.89	G → A/S
	301	2.00	154.58	0.09	−49.63	−47.56	A → P/S
	319	1.44	132.06	0.00	−57.45	−48.26	T → A/Q/–
	320	0.00	65.18	0.01	−26.93	−24.44	V → D/N/–
	462	1.08	12.87	0.08	−47.88	−45.03	V → I/T
RdRp of GII.P16	90	0.00	149.76	0.07	−10.82	−9.06	W → –
	117	2.07	29.83	0.09	−28.99	−25.65	T → V

### Emergence and migration of GII.P16-GII.2 norovirus in China

The animation shown in Supplementary Video 1 tracks the migration history of GII.P16-GII.2 norovirus between 2013 and 2018 in China and worldwide, revealing that GII.P16-GII.2 in China mainly came from Australia, the United States, Thailand, and Russia instead of neighboring Japan and South Korea. The earliest GII.P16-GII.2 norovirus strains were imported from Australia to Taiwan and from the United States to Guangdong, after which both domestic and foreign GII.P16-GII.2 norovirus disseminated throughout most areas of China. Domestically, there were roughly 3 epidemic centers that spread divergently to other parts of China: in the north, represented by Beijing; in the south, represented by Guangdong province and Shenzhen city; and in southeast coastal areas, represented by Zhejiang and Jiangsu provinces. Bayes modeling demonstrated a total of 20 well-supported dispersal routes (posterior probability >0.5), as summarized in Supplementary Table 3.

### Transmission reconstruction of GII.P16-GII.2 norovirus

**Fig. 2.**
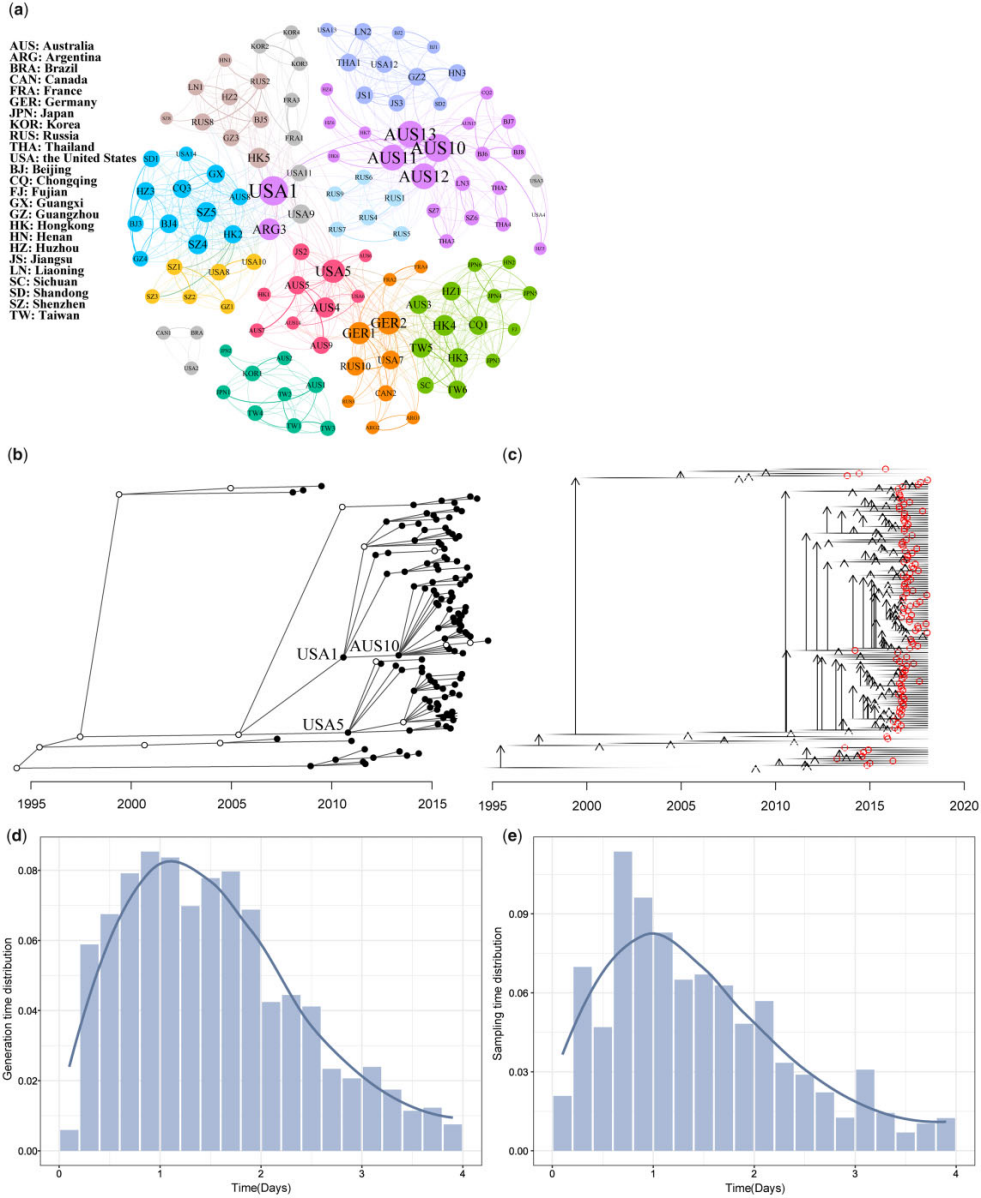
TransPhylo analysis of GII.P16-GII.2 norovirus. a) Modularity of the network structure, where color represents modularity and circle size represents the indegree of each strain. b) Consensus transmission tree, where empty circles represent unsampled cases and filled circles represent sample cases. The *x*-axis represents the infection date for each case, and the connection from 1 circle to another represents a transmission event. c) Detailed transmission tree, where each case (whether sampled or not) is shown as a horizontal line whose intensity is proportional to infectiousness. Vertical arrows and red circles represent transmission events. d) Realized generation time distribution (delay from infection to transmission) for individuals in the transmission tree. e) Realized sampling time distribution (delay from infection to sampling) for individuals in the transmission tree.

TransPhylo analyses showed that the basic reproductive number (*R_0_*), representing the number of secondary infections caused by each case, for GII.P16-GII.2 norovirus was 1.99 (95% CI: 1.58–2.44), and the sampling probability of each case was estimated to be 0.76 (95% CI: 0.63–0.88). A network analysis of transmission probability between cases, calculated using TransPhylo analysis, showed that the modularity of the network structure was 0.776 (Fig. 2a). The statistical parameters generated by Gephi, including indegree, outdegree, closeness centrality, betweenness centrality, and hub nodes, are summarized in Supplementary Table 4, which shows that the most highly exported strains were from the United States, Australia, and Germany, and the most imported strains were from Hong Kong and Shenzhen. Strains from the United States, Shenzhen, and Australia are hub nodes.

As shown in Fig. 2b, the outbreak of GII.P16-GII.2 norovirus was initiated with a single index case, with infection occurring before 1995, and the large-scale transmission of GII.P16-GII.2 norovirus started in 2010. USA1, USA5, and AUS10 caused relatively large transmissions. Compared with transmission events in recent years, early years were characterized by a limited number of infected cases in which 1 case usually infected 1 or 2 other patients. In later transmission chains, unsampled cases were also observed.

Figure 2c shows that most transmission occurred during high infectiousness (proportional to line intensity) and that most sampling (open red circles) occurred after transmission when infectiousness started to decline. Mean generation and sampling times, determined from the realized distributions of generation time (from infection to transmission) and sampling time (from infection to sampling) (Fig. 2, d and e), were approximately 1 and 0.76 days, respectively.

## Discussion

GII.P16-GII.2 norovirus was a rarely detected genotype before 2016, with only limited cases reported. However, epidemics caused by this genotype are currently increasing in China and elsewhere in the world. In this study, we performed comprehensive analyses of the molecular evolution and transmission of GII.P16-GII.2 norovirus. Our findings suggest that (1) this new variant may have evolved from GII.P16-GII.4 or previous GII.P16-GII.2 strains; (2) more positive selection sites were found in the *VP1* gene of GII.2 norovirus, possibly helping the virus to evade the host immune system and persist; (3) the earliest GII.P16-GII.2 norovirus in mainland China might have been imported from Australia to Taiwan or from the United States to Guangdong; and (4) the basic reproductive number (*R*_0_) of GII.P16-GII.2 norovirus was estimated to be 1.99 (95% CI: 1.58–2.44).

Norovirus shows high genetic diversity, and evolutionary and phylogenetic discrepancies between the *VP1* gene and *RdRp* region have frequently been reported (Nagasawa *et al.* 2018; Ozaki *et al.* 2018; Matsushima *et al.* 2019). One study found differences in the *VP1* gene and *RdRp* region of GII.P16-GII.2 norovirus and suggested that these 2 regions should be the focus of phylodynamic studies (Hernandez *et al.* 2018). Based on the GII.2 *VP1* gene, we estimated that the MRCA of GII.P16-GII.2 from China appeared in strains from Japan in 2001. That this coalescence dates to an earlier time may be explained by the absence of obvious differences from previous GII.P16-GII.2 strains circulating in 2011–2012, as supported by a study from China suggesting that this was not a novel recombinant but instead evolved from these previously circulating GII.P16-GII.2 strains (Tohma *et al.* 2017). GII.P16-GII.2, in turn, shared a MRCA with GII.P2-GII.2 in June 2000, as supported by a study from China reporting that the first reemerging GII.P16-GII.2 strain in China shared a MRCA with GII.P2-GII.2 (Ao *et al.* 2018). Based on the GII.P16 *RdRp* region, we estimated that the MRCA of GII.P16-GII.2 from China appeared in strains from Germany in 2014 and that GII.P16-GII.2 shared a MRCA with GII.P16-GII.4 strains from South Korea in February 2013. Considering the time at which GII.P16-GII.2 norovirus first reemerged in China in 2016–2017, the downward trend in GII.4 in Asia and worldwide since 2016 (Tohma *et al.* 2017; Van Beek *et al.* 2018), and the observation of more positive selection sites in the *VP1* gene, we speculate that the GII.P16-GII.2 strain likely evolved from GII.P16-GII.4. The findings of the current study are consistent with those of a study from Japan reporting that the reemergence of GII.P16-GII.2 norovirus might reflect a new recombination of GII.P16-GII.4 and the previous GII.P16-GII.2 (2010–2012 type) (Nagasawa *et al.* 2018). However, more positively selected sites found in *VP1* rather than *RdRp* could also be due to a higher degree of evolutionary constraints on amino acid change, thus allowing for greater sensitivity to detect adaptive change. Meanwhile, more neutral noise in *RdRp* reduces the power to detect adaptive signal. We found no obvious differences in evolutionary rates of the GII.2 *VP1* gene and GII.P16 *RdRp* region compared with those reported in previous studies. Both were estimated to be greater than 10^−3^ substitutions/site/year, but the GII.2 *VP1* gene evolves slower than the *RdRp* region, reflecting the fact that the capsid protein (*VP1*) has a more stable structure than the RNA polymerase (*RdRP*) and may play an important role in the evolutionary process of the norovirus (Lindesmith *et al.* 2012; Lu *et al.* 2016). About other parts of norovirus (*VP2*), we found that it is a less-studied HuNoV protein (Hassard *et al.* 2017) and only 1 reported that it evolved at a rate of 8.99 × 10^−3^ (95% CI: 6.59 × 10^−3^–11.6 × 10^−3^) in GII.4 not in GII.2 (Cotten *et al.* 2014).

Because phylogenetic analyses are unable to provide details on transmission between individuals, we applied a novel method, TransPhylo, to reconstruct transmission and extract several parameters of GII.P16-GII.2 norovirus detected in China and other countries. Two key parameters, *R*_0_ and pi, converged in the analysis, whereas neg diverged. The definition of neg is within-host coalescent rate, equaling the within-host effective population size (Ne) times generation duration g (days) divided by 365 (Didelot *et al.* 2021). Norovirus lives in the host for a short time, probably resulting small neg values that could not be estimated accurately. As expected, almost all unsampled cases occurred earlier, reflecting the inadequacies of surveillance and public health investigations at the time. At these earlier time points, 1 case usually infected 1 or 2 patients, whereas current cases are able to cause large outbreaks, indicating possible increased infectivity during the evolution of GII.P16-GII.2 norovirus. It is also possible that early sampling capacity was so weak that cases simply went undetected.

In China, the earliest GII.P16-GII.2 norovirus might have entered through 2 routes—1 from Australia to Taiwan and the other from the United States to Guangdong—rather than from Japan and South Korea, which is geographically closer to China and where more cases were found. Phylodynamics analyses of *VP1* and *RdRp* genomic regions showing that Chinese, Japanese, and South Korean strains were not divided into the same cluster but instead were neighboring clusters appeared to confirm this. This suggests that, because norovirus is highly contagious and can spread through contaminated water, air, food, and other items, geographic isolation is not a barrier to its spatial and temporal dispersal. Moreover, because this novel GII.P16-GII.2 norovirus spread from Guangdong to neighboring regions and northern China, including Zhejiang, Henan, Hong Kong, and Beijing, Guangdong may be the origin of reemergent GII.P16-GII.2 norovirus in China. Finally, our data suggest that GII.P16-GII.2 norovirus spread divergently from 3 epidemic centers—North (Beijing), South (Guangdong) and Southeast (Zhejiang and Jiangsu provinces)—to other parts of China. In these 3 areas, which are responsible for most exported cases, population mobility is a common feature, and Guangdong, Shenzhen, Zhejiang, and Jiangsu are coastal areas. Among them, Guangdong has been reported to be associated with the evolution and transmission of multiple genotypes of norovirus, including the first reemerging GII.P16-GII.2 norovirus (Lu *et al.* 2016; Ao *et al.* 2018). The reason why sporadic cases and community outbreaks of norovirus are common in coastal areas is that it has been often associated with the consumption of shellfish contaminated with fecal pollution (Campos and Lees 2014; Hassard *et al.* 2017). Overall, considering the infection risk, monitoring of shellfish safety and densely populated cities should be strengthened.

There are some limitations to this study. First, the sampling scheme was based on all available complete sequences that include origin country and sampling year, considering some cases were not sampled, which may bias origin and transmission results of GII.P16-GII.2 norovirus. Second, we excluded some sequences because they affect the temporal signal, perhaps it would be better to compare results using all included samples. Last but not the least, sequence quality, algorithms, and parameter settings could also bias the results because this study is mainly based on bioinformatics analyses. Although we used the constant size coalescent model after a selection process, perhaps using an exponential population model also is appropriate considering GII.P16-GII.2 suddenly appeared and became the main genotype. Finally, we recommend that there could be a study to reconstruct GII.P16-GII.2 evolution using smaller samples like outbreak within China. Overall, while there is a risk theoretically, we have discussed and compared results in our study with published articles mentioned above, so that we believe our results were reliable.

### Conclusions

Our results provide new insights into the early evolution and transmission of the reemerging GII.P16-GII.2 norovirus in China and worldwide. This reemerging strain may have evolved from GII.P16-GII.4 or previously circulating GII.P16-GII.2 strains, and the *VP1* gene might play a key role in this evolutionary process. Guangdong province is likely the origin of this reemerging GII.P16-GII.2 norovirus in China, and coastal and densely populated areas are responsible for its successful spread.

## Supplementary Material

jkac250_Supplemental_Material_Legends

jkac250_Supplementary_Figure_S1

jkac250_Supplementary_Figure_S2

jkac250_Supplementary_Table_S1

jkac250_Supplementary_Table_S2

jkac250_Supplementary_Table_S3

jkac250_Supplementary_Table_S4

jkac250_Supplementary_Video_S1

## Data Availability

Accession numbers of all sequences using in this study are provided in Supplementary Tables 1 and 2. Supplemental material is available at G3 online.
