# Supplementary material for: Early evolution and transmission of GII.P16-GII.2 norovirus in China
Source: G3 (Bethesda). 2022 Sep 19;12(11):jkac250. doi: 10.1093/g3journal/jkac250 (PMC9635637; doi:10.1093/g3journal/jkac250)
Supplement: jkac250_Supplemental_Material_Legends [file jkac250_supplemental_material_legends.docx]

**Supplementary material**

Fig S1. Trace plots of posterior values derived from 500,000 MCMC iterations with 10% burn-in generated by TansPhylo.

Fig S2. Colored phylogenetic tree combined phylogeney and transmission.

Table S1. GⅡ.2 VP1 and GⅡ.P16 RdRp complete sequences of norovirus for phylodynamic analysis.

Table S2. GⅡ.P16-GⅡ.2 complete sequences of norovirus for phylogeography analysis.

Table S3. Well-supported dispersal routes (posterior probability > 0.5) of GⅡ.P16-GⅡ.2 norovirus generated by phylogeography analysis.

Table S4. Statistical parameters calculated by Gephi software.

Video S1. Early diffusion process of GⅡ.P16-GⅡ.2 norovirus in China and worldwide.
