# Supplementary material for: Early evolution and transmission of GII.P16-GII.2 norovirus in China
Source: G3 (Bethesda). 2022 Sep 19;12(11):jkac250. doi: 10.1093/g3journal/jkac250 (PMC9635637; doi:10.1093/g3journal/jkac250)
Supplement: jkac250_Supplementary_Figure_S1 [file jkac250_supplementary_figure_s1.docx]

**

**

Figure S1. Trace plots of posterior values derived from 500,000 MCMC iterations with 10% burn-in generated by TransPhylo.
