# Supplementary material for: Early evolution and transmission of GII.P16-GII.2 norovirus in China
Source: G3 (Bethesda). 2022 Sep 19;12(11):jkac250. doi: 10.1093/g3journal/jkac250 (PMC9635637; doi:10.1093/g3journal/jkac250)
Supplement: jkac250_Supplementary_Figure_S2 [file jkac250_supplementary_figure_s2.docx]

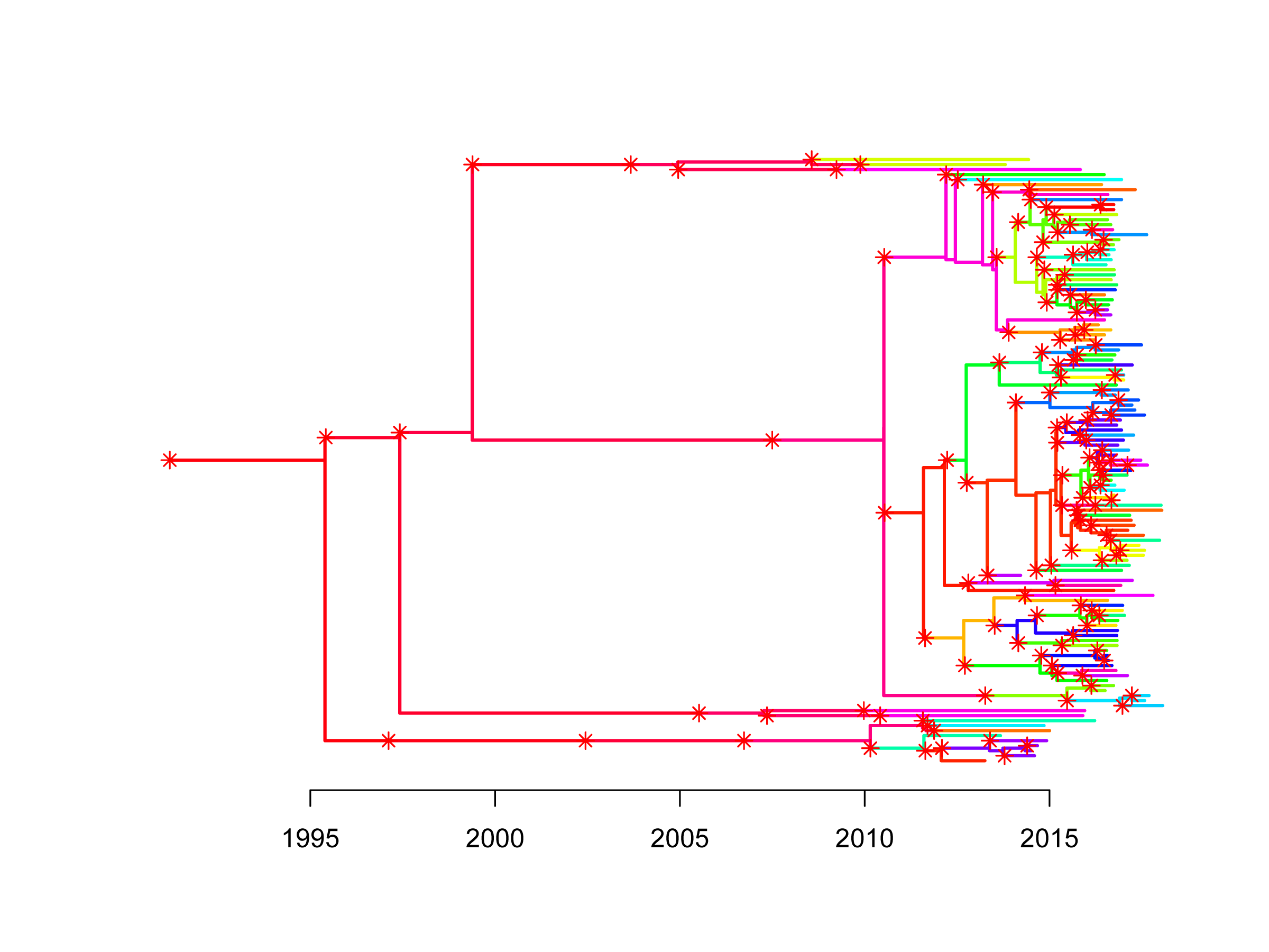


Figure S2. Colored phylogenetic tree combined phylogeny and transmission, where each host is represented by a unique color. A transmission event happened from one color to another color, and these are highlighted with red stars.
